# Incidence, Risk Factors and Prognosis of T4a Gastric Cancer: A Population-Based Study

**DOI:** 10.3389/fmed.2021.767904

**Published:** 2022-01-05

**Authors:** Zhiya Hu, Ziyi Zuo, Han Miao, Zhijie Ning, Youyuan Deng

**Affiliations:** ^1^Department of Pharmacy, The Third Hospital of Changsha, Changsha, China; ^2^The First Clinical College, Wenzhou Medical University, Wenzhou, China; ^3^Department of Clinical Medicine, Dalian University, Dalian, China; ^4^Department of Neurology, Fengcheng Hospital, Shanghai, China; ^5^Department of General Surgery, Xiangtan Central Hospital, Xiangtan, China

**Keywords:** nomogram, T4a gastric cancer, prognosis, overall survival, cancer-specific survival, SEER

## Abstract

**Background:** T4a gastric cancer (GC) is a subtype of advanced GC (AGC), which urgently needs a comprehensive grade method for better treatment strategy choosing. The purpose of this study was to develop two nomograms for predicting the prognosis of patients with T4a GC.

**Methods:** A total of 1,129 patients diagnosed as T4a GC between 2010 and 2015 were extracted from the Surveillance, Epidemiology, and End Result (SEER) program database. Univariate and multivariate Cox analyses were performed to explore the independent predictors and to establish nomogram for overall survival (OS) of the patients, whereas competing risk analyses were performed to find the independent predictors and to establish nomogram for cancer-specific survival (CSS) of the patients. The area under the curve (AUC), calibration curve, decision curve analysis (DCA), and Kaplan–Meier analysis were performed to evaluate the nomograms.

**Results:** Older age, larger tumor size, black race, signet ring cell carcinoma (SRCC), more lymph node involvement, the absence of surgery, the absence of radiotherapy, and the absence of chemotherapy were identified as independent prognostic factors for both OS and CSS. In the training cohort, the AUCs of the OS nomogram were 0.760, 0.743, and 0.723 for 1-, 3-, and 5-year OS, whereas the AUCs of the CSS nomogram were 0.724, 0.703, and 0.713 for 1-, 3-, and 5-year CSS, respectively. The calibration curve and DCA indicated that both nomograms can effectively predict OS and CSS, respectively. The abovementioned results were also confirmed in the validation cohort. Stratification of the patients into high- and low-risk groups highlighted the differences in prognosis between the two groups both in training and in validation cohorts.

**Conclusions:** Age, tumor size, race, histologic type, N stage, surgery status, radiotherapy, and chemotherapy were confirmed as independent prognostic factors for both OS and CSS in patients with T4a GC. Two nomograms based on the abovementioned variables were constructed to provide more accurate individual survival predictions for them.

## Introduction

Gastric cancer (GC) is the fifth malignancy and ranks third in cancer-related mortality worldwide (1). Advanced gastric cancer (AGC) is a common type of GC with a poor prognosis, and the 5-year survival rate is <20% (2, 3). Even if radical resection, the 5-year survival rate is only 30–50% (4). Currently, neoadjuvant therapy and radical resection combined with chemotherapy have been systematically used for patients with AGC (5). However, due to the high risk of distant metastasis, the survival of these patients was still unsatisfactory.

As an important branch of patients with AGC, patients with T4a GC have their unique characteristics. According to the 8th version of the TNM staging system of the American Joint Committee on Cancer (AJCC), T4a GC was defined as the tumor perforating serosa (6). Owing to the presence of incurable factors including distant lymph node involvement, peritoneal metastasis, and hematogenous metastasis, the outcomes of traditional treatments varied distinctly in this group of patients (7, 8). Further, with the complexity of the prognosis and its influencing factors, there are still challenges in assessing the prognosis precisely and individually. Therefore, an effective grading and stratification system of T4a GC is of great importance for treatment choosing prior to operation to have a higher chance of curative resection and reduce postoperative morbidity and mortality (9). However, for T4a GC, the relevant study is still lacking.

The nomogram is a practical tool in medical practice, which could pictorially represent a multivariable model. Recently, nomograms mostly concentrate on the patients of early-stage or under single treatment, and they mostly adequate for prognosis predicting intraoperatively or postoperatively (10–16). However, there still few nomograms integrating all the relevant clinicopathologic characters for patients with T4a GC. In this study, based on the Surveillance, Epidemiology, and End Result (SEER) program database, we aimed to identify the prognostic factors of patients with T4a GC and develop nomograms to predict overall survival (OS) and cancer-specific survival (CSS).

## Materials and Methods

### Patients

All patients with first primary GC between 2010 and 2015 with SEER Stat 3.6.1 were included. The SEER database is publicly available without any personal information. The inclusion criteria were as follows: (1) first primary T4a GC and (2) follow up greater than or equal to 1 month. Meanwhile, patients without clear baseline information, tumor characteristics, and treatment data were excluded. Patients who meet the abovementioned criteria were randomly divided into the training set (70%) and the validation set (30%). In this study, the nomograms were constructed based on the training set and were validated by the validation set.

### Variables

The variables utilized in this study were age at diagnosis, sex, race, histologic type, tumor size, tumor grade, N stage, marriage status, insurance status, surgery, radiotherapy, and chemotherapy. Age and tumor size were transferred into categorical variables, and the cutoff values were calculated by X-tile software (17). In this study, age and tumor size were divided into low, medium, and high levels. N stage was described as N0, N1, N2, and N3. Tumor grade was classified as well differentiated (I), moderately differentiated (II), poorly differentiated (III), and undifferentiated anaplastic (IV). In this study, OS and CSS were considered as outcomes. The OS was defined as the time interval from the date of primary diagnosis to the date of death caused by any cause, and the CSS was defined as the time interval from the date of primary diagnosis to the date of GC-specific death.

### Statistical Analysis

The univariate and multivariate Cox analyses were performed to explore the independent prognostic factors and establish the nomogram for predicting the OS of patients with T4a GC, whereas competing risk analyses were performed to find the independent predictors and to establish nomogram for CSS of the patients. Besides, the time-dependent receiver operating characteristic (ROC) curve for the nomograms was established, and the area under the curve (AUC) was calculated to show the discrimination of the nomograms. Calibration curves were established to compare the probability between nomogram-predicted and observed outcomes, and the decision curve analysis (DCA) was used to explore the clinical utilization of the nomograms. Further, we categorized the patients into high- and low-risk groups according to their median risk score, survival analysis was performed to probe the difference in prognosis between the two groups using the Kaplan–Meier method, and the log-rank test was performed. Moreover, two nomograms were verified with the validation cohort. In this study, all statistical analyses were performed with SPSS 25.0 and R software (version 3.6.1), and a *p* < 0.05 (two-tailed) was considered statistically significant.

## Results

### Baseline Patient Demographics

In our study, to explore the difference in clinicopathological factors between patients with T4a and other GC, all the clinicopathological data of patients with GC were extracted and comparison showed a distinct difference between patients with T4a and other GC in all the variables included in this study (Table 1). Besides, a total of 1,129 patients with T4a GC were included and were randomly divided into the training cohort (*n* = 793) and the validation cohort (*n* = 336). The baseline demographics and clinicopathologic characteristics are listed in Table 2. The optimal cutoff value of tumor size and age at diagnosis were calculated separately for OS and CSS. Tumor size was divided into ≤31 mm, 32–90 mm, and ≥91 mm based on OS, and the same grouping was also concluded based on CSS. Moreover, the optimal cutoff value of age at diagnosis was identified as 65 and 80 years based on OS status and CSS.

**Table 1:** Clinical characteristics between patients with T4a and non-T4a GC.

**Variables**		**T4a GC**	**Non-T4a GC**	***p***
		***n* = 1,446 (%)**	***n* = 8,292 (%)**	
Age [mean (SD)]		64.69 (14.18)	66.16 (13.15)	**<0.001**
Race				**<0.001**
	White	901 (62.31)	5,713 (68.9)	
	Black	192 (13.28)	1,014 (12.23)	
	Other	353 (24.41)	1,565 (18.87)	
Sex				**<0.001**
	Female	632 (43.71)	2,775 (33.47)	
	Male	814 (56.29)	5,517 (66.53)	
Histologic type				**<0.001**
	Adenocarcinoma	974 (67.36)	6,750 (81.4)	
	Mucinous adenocarcinoma	52 (3.6)	184 (2.22)	
	Signet ring cell carcinoma	420 (29.05)	1,358 (16.38)	
Tumor size				**<0.001**
	Low	247 (17.08)	3,391 (40.89)	
	Middle	973 (67.29)	4,348 (52.44)	
	High	226 (15.63)	553 (6.67)	
Grade				**<0.001**
	I	14 (0.97)	552 (6.66)	
	II	235 (16.25)	2,672 (32.22)	
	III	1,161 (80.29)	4,915 (59.27)	
	IV	36 (2.49)	153 (1.85)	
AJCC stage				**<0.001**
	I	0 (0)	2,455 (29.61)	
	II	174 (12.03)	2,000 (24.12)	
	III	955 (66.04)	2,430 (29.31)	
	IV	317 (21.92)	1,407 (16.97)	
N				**<0.001**
	N0	244 (16.87)	3,815 (46.01)	
	N1	284 (19.64)	2,385 (28.76)	
	N2	300 (20.75)	1,116 (13.46)	
	N3	618 (42.74)	976 (11.77)	
M				**<0.001**
	M0	1,129 (78.08)	6,885 (83.03)	
	M1	317 (21.92)	1,407 (16.97)	
Insurance				**0.017**
	No	60 (4.15)	246 (2.97)	
	Yes	1,386 (95.85)	8,046 (97.03)	
Marriage				**0.009**
	No	588 (40.66)	3,071 (37.04)	
	Yes	858 (59.34)	5,221 (62.96)	

*Bold values indicate the corresponding variable have statistical significance (P < 0.05)*.

**Table 2:** Baseline information of 1,129 patients T4aN0-3M0 gastric cancer.

		**Whole cohort (*n* = 1,129)**	**Training cohort (*n* = 793)**	**Validation cohort (*n* = 336)**
Age		65.65 ± 14.01	65.34 ± 14.01	66.37 ± 14.02
Race				
	Black	154 (13.64%)	113 (14.25%)	41 (12.20%)
	White	694 (61.47%)	482 (60.78%)	212 (63.10%)
	Other	281 (24.89%)	198 (24.97%)	83 (24.70%)
Sex				
	Female	500 (44.29%)	345 (43.51%)	155 (46.13%)
	Male	629 (55.71%)	448 (56.49%)	181 (53.87%)
Histologic type				
	Adenocarcinoma	754 (66.78%)	537 (67.72%)	217 (64.58%)
	Mucinous adenocarcinoma	34 (3.01%)	24 (3.03%)	10 (2.98%)
	Signet ring cell carcinoma	341 (30.20%)	232 (29.26%)	109 (32.44%)
Tumor size		62.43 ± 43.50	63.09 ± 46.54	60.88 ± 35.34
Grade				
	I	12 (1.06%)	9 (1.13%)	3 (0.89%)
	II	173 (15.32%)	126 (15.89%)	47 (13.99%)
	III	920 (81.49%)	641 (80.83%)	279 (83.04%)
	IV	24 (2.13%)	17 (2.14%)	7 (2.08%)
N stage				
	N0	196 (17.36%)	137 (17.28%)	59 (17.56%)
	N1	219 (19.40%)	161 (20.30%)	58 (17.26%)
	N2	246 (21.79%)	170 (21.44%)	76 (22.62%)
	N3	468 (41.45%)	325 (40.98%)	143 (42.56%)
Surgery performed		1,076 (95.31%)	748 (94.33%)	328 (97.62%)
Radiotherapy performed		466 (41.28%)	321 (40.48%)	145 (43.15%)
Chemotherapy performed		751 (66.52%)	518 (65.32%)	233 (69.35%)
Insurance		1,089 (96.46%)	762 (96.09%)	327 (97.32%)
Married		664 (58.81%)	464 (58.51%)	200 (59.52%)

### Identification of Prognosis Factors

The results of the univariate analysis for predicting OS of patients with T4a GC in the training cohort are shown in Table 3; the significant OS-related variables were age, race, tumor size, N stage, grade, histologic type, marriage status, surgery, radiotherapy, and chemotherapy. These factors were further included in the multivariate Cox analysis. Finally, age, tumor size, race, N stage, surgery status, radiotherapy, and chemotherapy were identified as independent prognostic factors for OS in patients with T4a GC. Moreover, to facilitate the clinical utilities of the model, we added the variable of histological type into the final model for its biological plausibility. In addition, the same analyses were reperformed in the whole cohort, and we concluded the same conclusions as mentioned above (Supplementary Table 1).

**Table 3:** Univariate and multivariate Cox analyses for the OS of patients with T4aN0-3M0 GC in the training cohort.

		**Univariate Cox**			**Multivariate Cox**		
		**HR**	**95% CI**	***p-value***	**HR**	**95%CI**	***p*-value**
Age							
	Low	Reference					
	Middle	**1.311**	**1.085–1.583**	**0.005**	**1.332**	**1.098–1.615**	**0.004**
	High	**1.896**	**1.508–2.382**	**0.000**	**1.561**	**1.216–2.005**	**0.000**
Race							
	White	Reference					
	Black	0.981	0.767–1.254	0.876	1.101	0.855–1.416	0.456
	Other	**0.760**	**0.617–0.936**	**0.010**	**0.702**	**0.568–0.867**	**0.001**
Sex (male)		1.026	0.866–1.216	0.765			
Histologic type							
	Adenocarcinoma	Reference					
	Mucinous adenocarcinoma	0.833	0.479–1.449	0.518	0.815	0.465–1.426	0.473
	Signet ring cell carcinoma	**1.251**	**1.044–1.498**	**0.015**	1.162	0.958–1.409	0.127
Size							
	Low	Reference					
	Middle	**1.323**	**1.043–1.678**	**0.021**	1.130	0.883–1.447	0.332
	High	**2.064**	**1.543–2.762**	**0.000**	**1.625**	**1.195–2.209**	**0.002**
Grade							
	I	Reference					
	II	1.200	0.483–2.981	0.694	0.818	0.326–2.051	0.669
	III	1.986	0.822–4.801	0.128	1.353	0.552–3.318	0.509
	IV	**3.126**	**1.134–8.619**	**0.028**	1.882	0.672–5.274	0.229
N stage							
	N0	Reference					
	N1	1.241	0.926–1.662	0.148	**1.360**	**1.008–1.836**	**0.044**
	N2	1.220	0.911–1.633	0.182	**1.461**	**1.082–1.972**	**0.013**
	N3	**1.880**	**1.460–2.421**	**0.000**	**2.035**	**1.550–2.670**	**0.000**
Surgery performed		**0.398**	**0.292–0.543**	**0.000**	**0.294**	**0.212–0.407**	**0.000**
Radiotherapy performed		**0.609**	**0.511–0.726**	**0.000**	**0.750**	**0.611–0.920**	**0.006**
Chemotherapy performed		**0.551**	**0.464–0.655**	**0.000**	**0.591**	**0.476–0.733**	**0.000**
Insurance (yes)		0.820	0.540–1.245	0.351			
Married (yes)		**0.815**	**0.688–0.966**	**0.019**	0.961	0.803–1.150	0.664

*Bold values indicate the corresponding variable have statistical significance (P < 0.05)*.

For identifying prognostic factors related to CSS of patients with T4a GC, multivariate competing risk model analyses were performed, and age, tumor size, race, N stage, surgery status, radiotherapy, and chemotherapy were identified as independent prognostic factors for CSS in patients with T4a GC (Table 4). Moreover, to facilitate the clinical utilities of the model, we added the variable of histological type into the model for its biological plausibility. In addition, the same analyses were reperformed in the whole cohort, and we concluded the same conclusions as mentioned above (Supplementary Table 2).

**Table 4:** Multivariate competing risk model analysis of CSS for each variable in T4aN0-3M0 patients with T4aN0-3M0 GC in the training cohort.

		**HR**	**95% CI**	***p***
Age			
	Low		Reference	
	Middle	1.126	0.917–1.382	0.260
	High	**1.330**	**1.001–1.767**	**0.049**
Race				
	White		Reference	
	Black	0.995	0.758–1.305	0.970
	Other	**0.676**	**0.541–0.845**	**0.001**
Sex (male)		1.078	0.894–1.300	0.430
Histologic type			
	Adenocarcinoma		Reference	
	Mucinous adenocarcinoma	0.705	0.371–1.341	0.290
	Signet ring cell carcinoma	1.191	0.968–1.466	0.099
Size			
	Low		Reference	
	Middle	1.136	0.889–1.451	0.310
	High	**1.582**	**1.151–2.173**	**0.005**
Grade			
	I		Reference	
	II	0.932	0.343–2.527	0.890
	III	1.471	0.556–3.893	0.440
	IV	2.475	0.857–7.149	0.094
N stage			
	N0		Reference	
	N1	1.360	0.980–1.889	0.066
	N2	**1.561**	**1.134–2.148**	**0.006**
	N3	**1.972**	**1.472–2.642**	**0.000**
Surgery performed		**0.341**	**0.230–0.504**	**0.000**
Radiotherapy performed		**0.699**	**0.571–0.856**	**0.001**
Chemotherapy performed		**0.667**	**0.530–0.839**	**0.001**
Insurance (yes)		0.798	0.494–1.289	0.360
Married (yes)		0.933	0.764–1.139	0.490

*Bold values indicate the corresponding variable have statistical significance (P < 0.05)*.

### Construction of Nomograms

**Figure 1:**
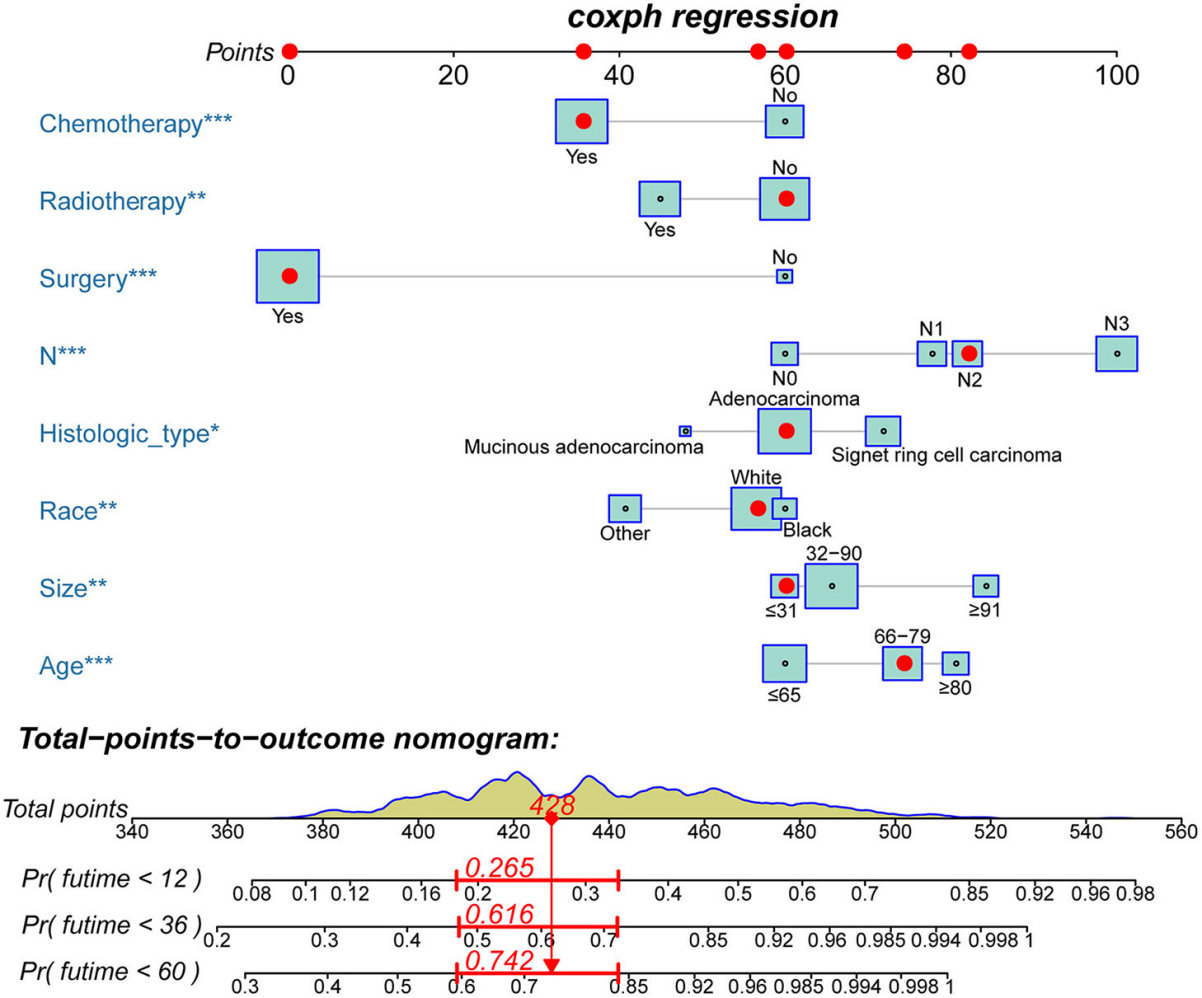
The novel nomogram to predict 1-, 3-, and 5-year OS of patients with T4a GC.

**Figure 2:**
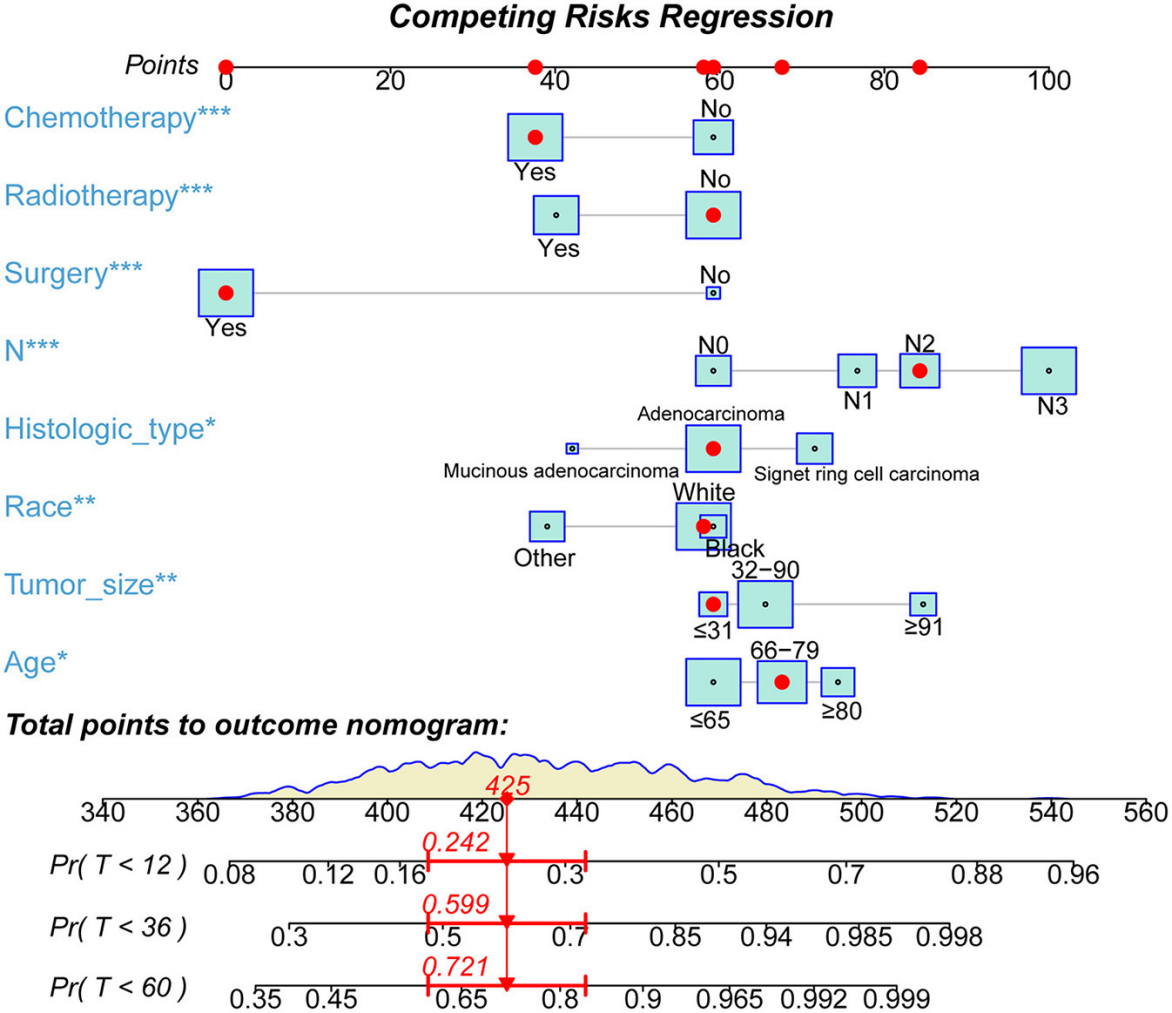
The novel nomogram to predict 1-, 3-, and 5-year CSS of patients with T4a GC.

**Figure 3:**
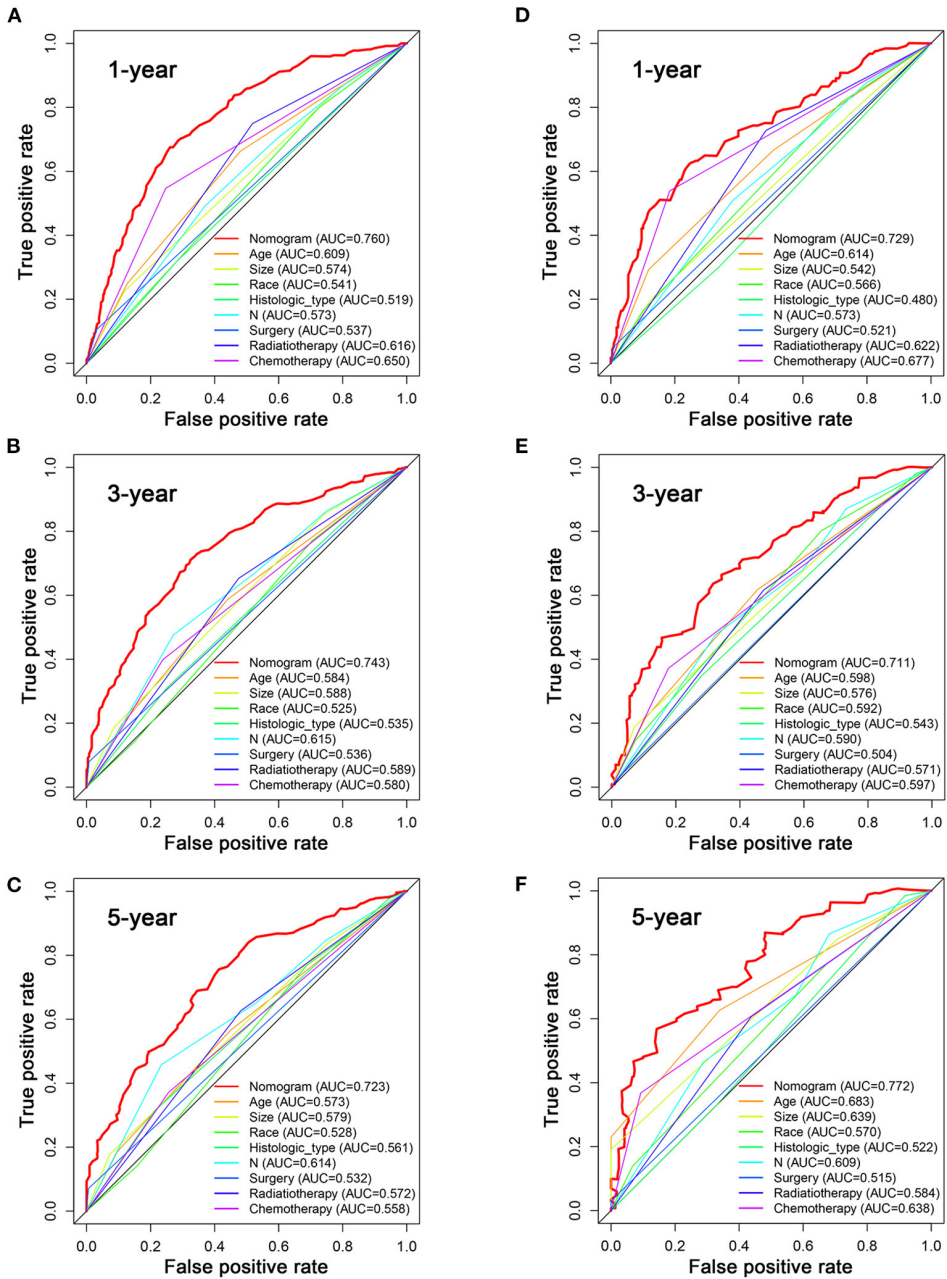
The ROC curves for predicting patients' OS of nomogram and all independent predictors at 1 **(A)**, 3 **(B)**, and 5 years **(C)** in the training cohort and at 1 **(D)**, 3 **(E)**, and 5 years **(F)** in the validation cohort.

**Figure 4:**
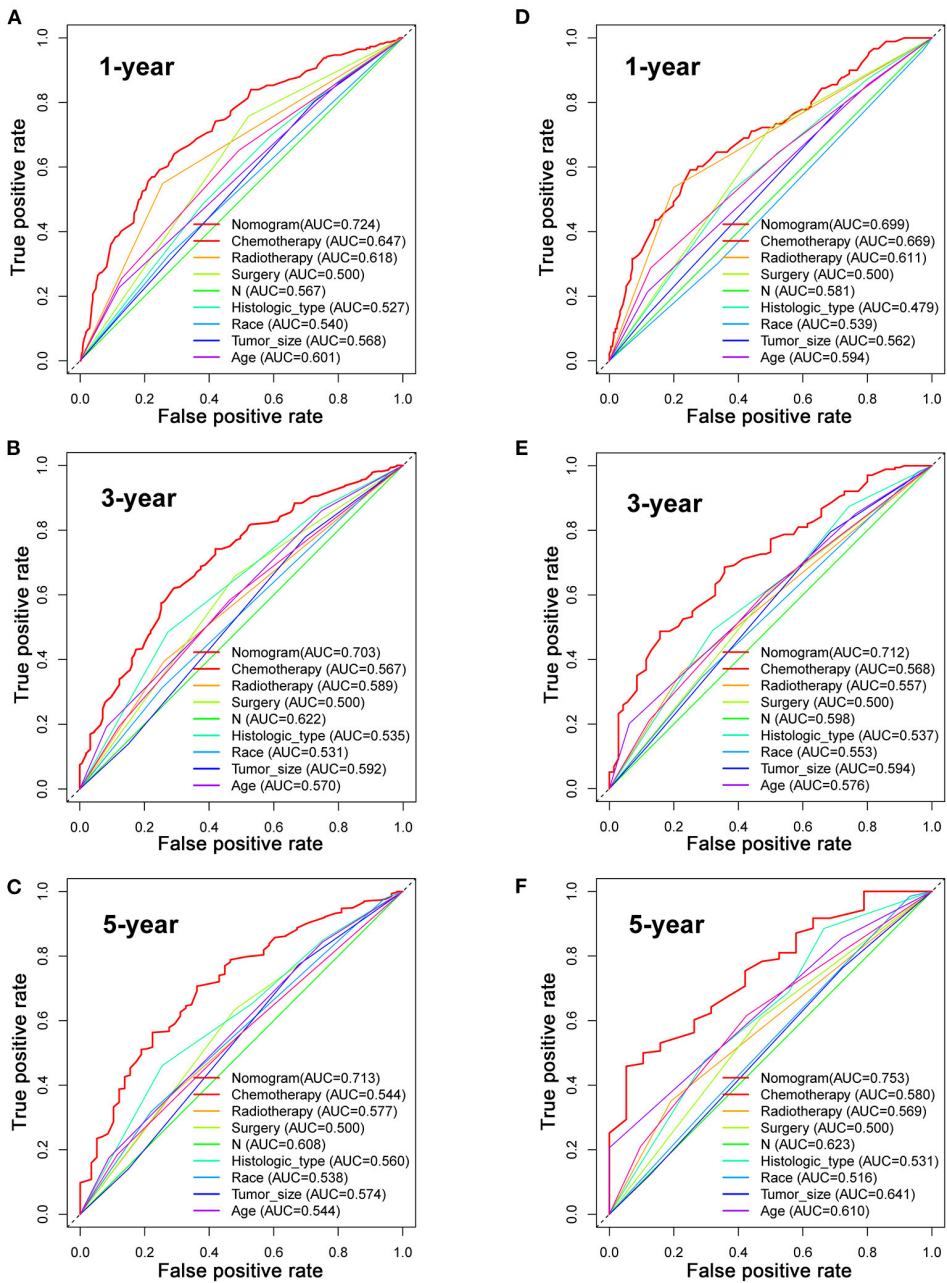
The ROC curves for predicting patients' CSS of nomogram and all independent predictors at 1 **(A)**, 3 **(B)**, and 5 years **(C)** in the training cohort and at 1 **(D)**, 3 **(E)**, and 5 years **(F)** in the validation cohort.

**Figure 5:**
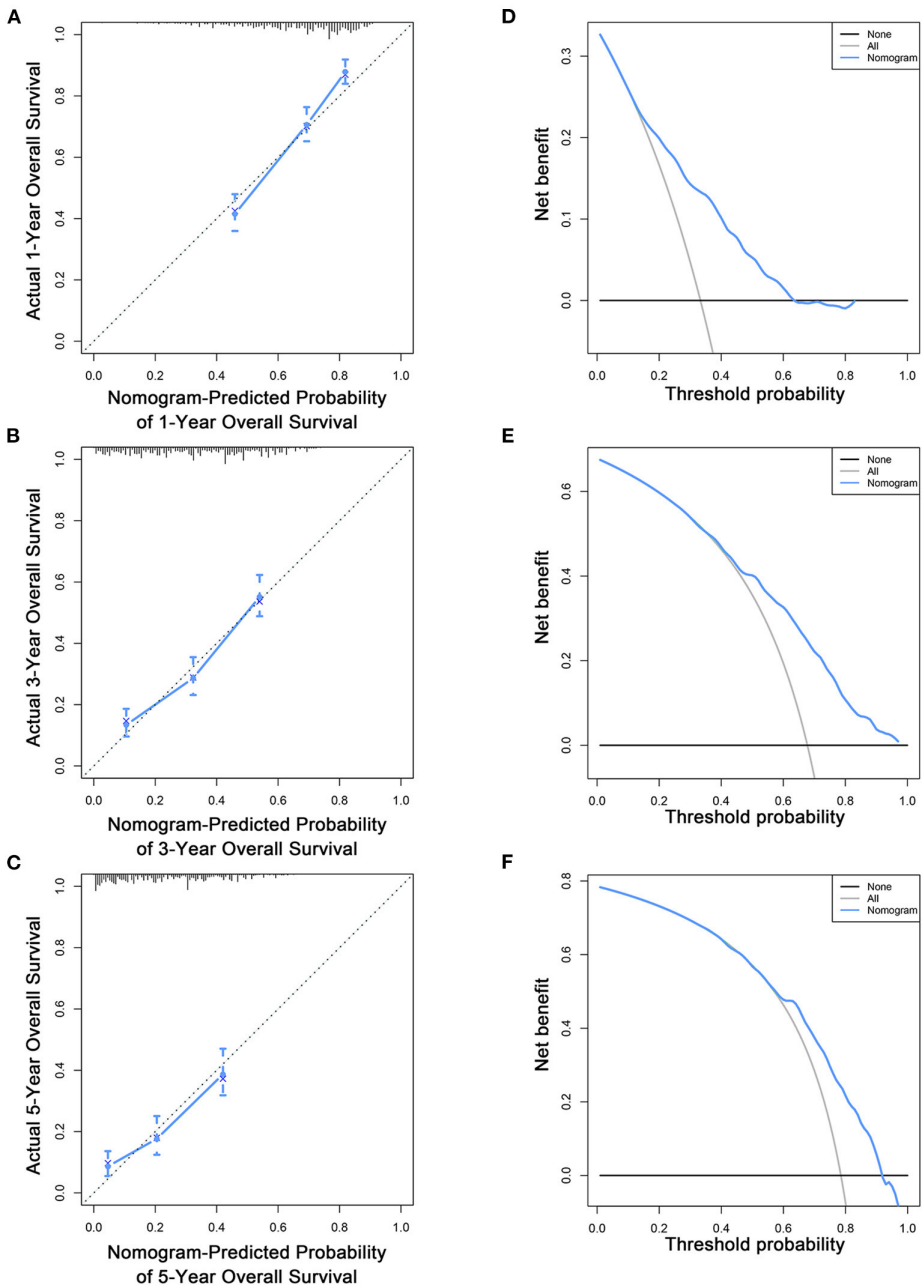
Calibration curves **(A–C)** and DCA **(D–F)** of the nomogram for predicting patients' OS at 1, 3, and 5 years in the training cohort.

**Figure 6:**
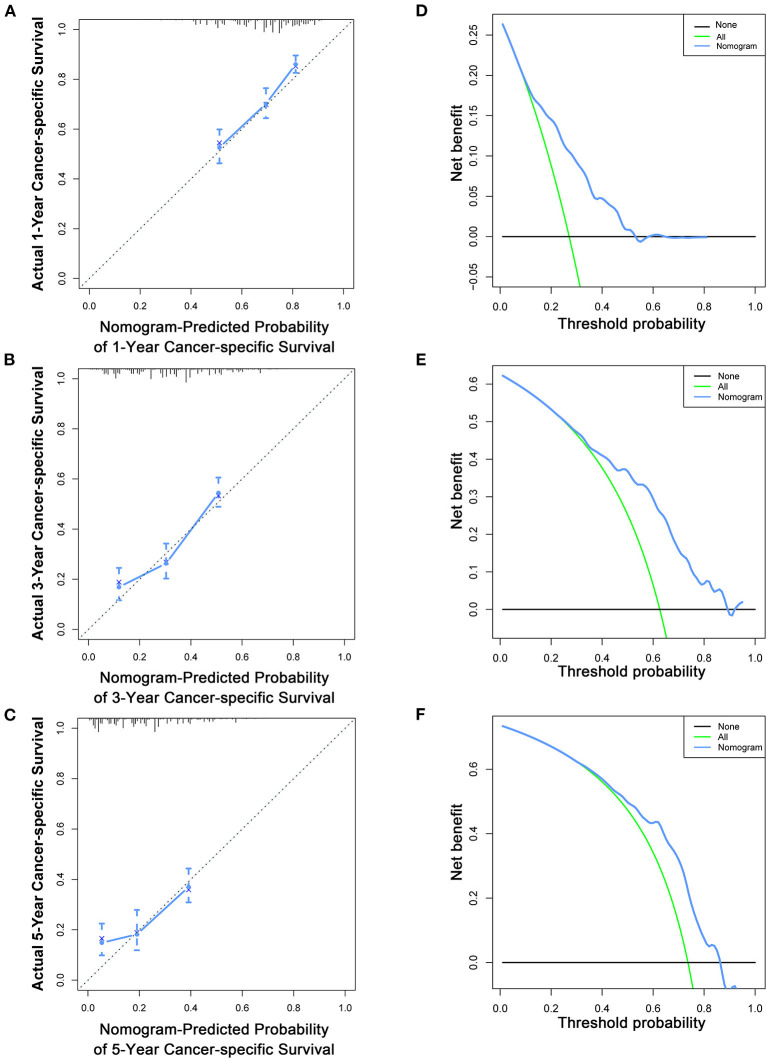
Calibration curves **(A–C)** and DCA **(D–F)** of the nomogram for predicting patients' CSS at 1, 3, and 5 years in the training cohort.

Based on the multivariate Cox analysis and multivariate competing risk model, two nomograms to predict 1-, 3-, and 5-year OS and CSS that integrated the abovementioned independent factors were conducted (Figures 1, 2). As for these two nomograms, we can obtain the corresponding survival probability of each patient by adding up all points that correspond to each predictor. The ROC curve demonstrated the discrimination of the nomograms. The AUCs of the nomogram for predicting 1-, 3-, and 5-year OS were 0.760, 0.743, and 0.723, respectively (Figures 3A–C). The AUCs of the nomogram that predicting 1-, 3-, and 5-year CSS were 0.724, 0.703, and 0.713, respectively (Figures 4A–C). The calibration curves of OS (Figures 5A–C) and CSS (Figures 6A–C) showed optimal predictive accuracy. Moreover, DCA of 1-, 3-, and 5-year OS (Figures 5D–F) and CSS (Figures 6D–F) showed that the nomograms had a higher net benefit in the training cohort which indicated a favorable clinical utilization.

### Validation of the Nomograms

**Figure 7:**
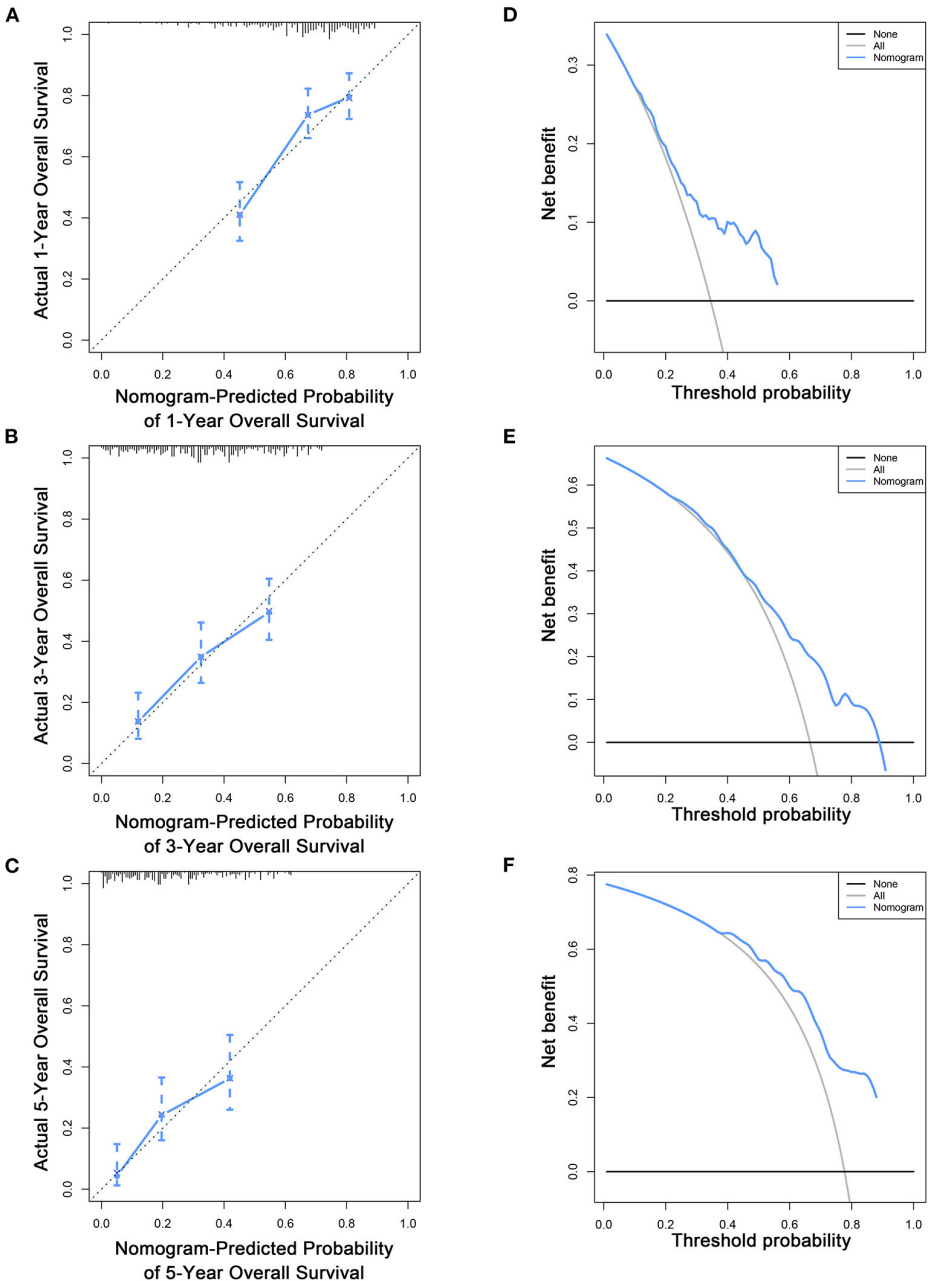
Calibration curves **(A–C)** and DCA **(D–F)** of the nomogram for predicting patients' OS at 1, 3, and 5 years in the validation cohort.

**Figure 8:**
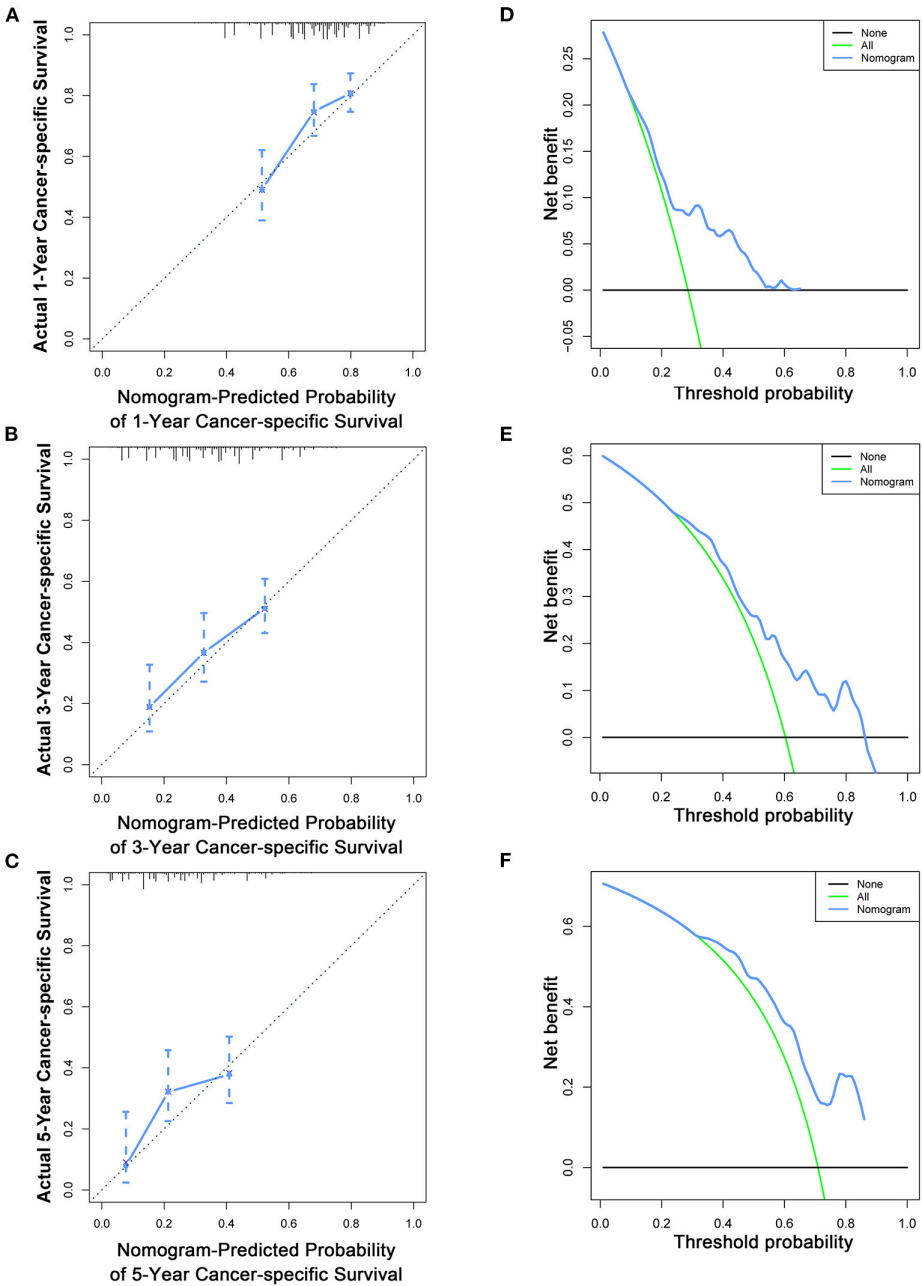
Calibration curves **(A–C)** and DCA **(D–F)** of the nomogram for predicting patients' CSS at 1, 3, and 5 years in the validation cohort.

To estimate the performance of the nomograms, external validation was performed, and the results demonstrated that the nomograms also have favorable outcomes in the validation cohort. The AUCs of the nomogram for predicting 1-, 3-, and 5-year OS were 0.729, 0.711, and 0.772, respectively (Figures 3D–F). The AUCs of the nomogram that predicting 1-, 3-, and 5-year CSS were 0.699, 0.712, and 0.753, respectively (Figures 4D–F). The calibration curves for the OS (Figures 7A–C) and CSS (Figures 8A–C) probabilities further validated the nomograms. Moreover, DCA of 1-, 3-, and 5-year OS (Figures 7D–F) and CSS (Figures 8D–F) also confirmed that the nomograms have favorable clinical utilization in the validation cohort.

### Risk Stratification for Patients With T4a GC

**Figure 9:**
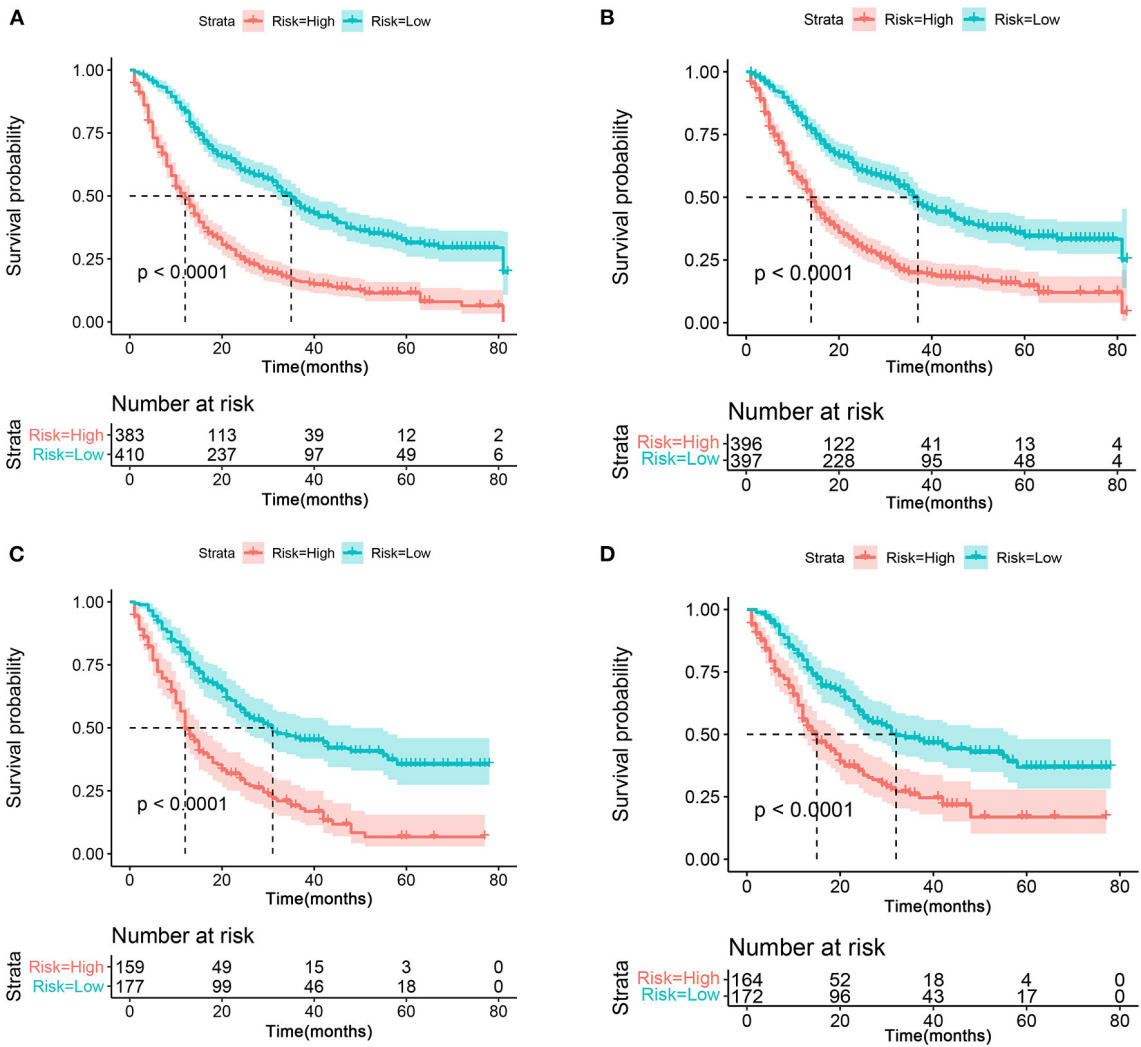
The Kaplan–Meier survival curve of risk group stratification for OS **(A)** and CSS **(B)** in the training cohort. The Kaplan–Meier survival curve of risk group stratification for OS **(C)** and CSS **(D)** in the validation cohort.

As mentioned above, for patients with T4a GC, risk stratification is of great importance in downgrading the GC prior to resection, thus improving the treatment management and the chance of curative resection. Therefore, we further stratified the patient into high- and low-risk groups based on the median risk score. Kaplan–Meier survival analysis showed favorable OS and CSS in the low-risk group compared with the high-risk group (Figures 9A,B). In the validation cohort, a favorable prognosis was also observed in the low-risk group for both OS and CSS (Figures 9C,D).

### Comparison Between the Novel Nomograms and AJCC Stage System

**Figure 10:**
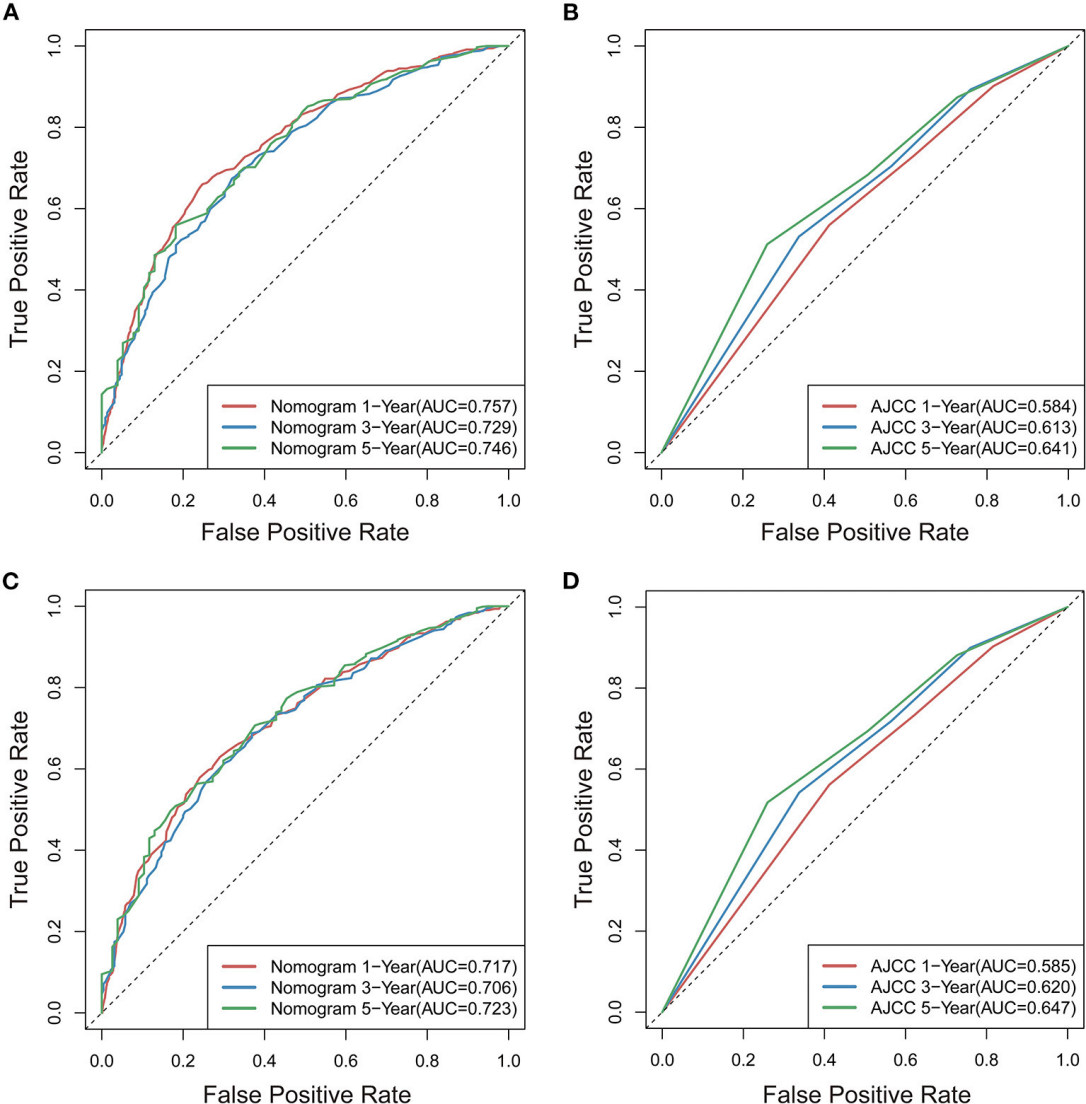
Comparison of ROC curves for predicting patients' OS at 1, 3, and 5years between novel nomogram **(A)** and AJCC model **(B)**, for predicting patients' CSS at 1, 3, and 5years between novel nomogram **(C)** and AJCC model **(D)**.

To further estimate the performance and clinical value of the nomograms in this study, we further compared the nomograms with AJCC stage system in the whole study cohort using ROC curve analysis, and results show that the nomograms also have favorable outcomes compared with AJCC stage system. The AUCs of the nomogram for predicting 1-, 3-, and 5-year OS were 0.757, 0.729, and 0.746, respectively (Figure 10A), whereas the AUCs of AJCC stage system were 0.584, 0.613, and 0.641, respectively (Figure 10B). The AUCs of the nomogram for predicting 1-, 3-, and 5-year CSS were 0.717, 0.706, and 0.723, respectively (Figure 10C), whereas the AUCs of AJCC stage system were 0.585, 0.620, and 0.647 (Figure 10D), separately.

## Discussion

In this study, older age, larger tumor size, black race, signet ring cell carcinoma (SRCC), more lymph node involvement, the absence of surgery, the absence of radiotherapy, and the absence of chemotherapy were found to be negatively associated with both OS and CSS in patients with T4a GC. Based on that, two nomograms for predicting the 1-, 3-, and 5-year OS and CSS of patients with T4a GC were constructed and validated. Discrimination, calibration, and clinical utilization analysis further confirmed that the nomograms we constructed were of favorable effectiveness and accuracy. The proposed nomograms also showed favorable ability to categorize patients with T4a GC into high- and low-risk groups with significant differences in OS and CSS.

As described before, owing to the presence of incurable factors such as distant lymph node involvement, peritoneal metastasis, and hematogenous metastasis, patients with T4a GC always suffered from distinctly different outcomes of traditional treatments (7, 8). In this study, we compared the clinicopathological factors and also other factors between patients with T4a and other GC and found T4a GC always accompanied with larger tumor size, more severe tumor grade, more lymph node invasion, and distant metastasis, which may lead to a worse clinical outcome. Moreover, with the complexity of the prognosis and its influencing factors, there are still challenges in assessing the prognosis precisely and individually. Therefore, this study paved a new way in assessing the patients' potential risk under different clinicopathological backgrounds and different medical procedures. Based on the validations and the comparison between models that widely used, we hope that the novel nomograms could better assist the clinical practice.

To date, there are many nomograms for GC, and they have shown clinical utilization from different aspects. In the postoperative perspective, the nomogram from Memorial Sloan-Kettering Cancer Center (MSKCC) was conducted to predict the survival probability of patients with GC after an R0 resection (14). From the intraoperative perspective, the PMN stage system was introduced recently to make a better treatment decision for patients with AGC (13). In addition to these studies, there also have different nomograms that integrated biomarkers (10) and other clinical characters (11). Recently, collagen nomograms have provided a new direction to evaluate the recurrence and metastasis of GC (18); moreover, with the continuous progress of radiomics, nomograms based on radiomics signatures also paved a new way for the diagnosis and evaluation of GC (12).

Compared with the abovementioned studies, our nomograms have several improvements. First, most of the studies were focused on the survival status of whole patients with GC or AGC (10–16, 18). Instead, we went a step further and focused on T4a GC in the subdivided perspective. Owing to the complexity of the prognosis and its influencing factors, a precision stratification is crucial to apply individual and also neoadjuvant therapies to downgrade the GC prior to surgery thus enhancing the opportunity for a curative resection (9) and maximize the benefits of patients. So, we integrated various prognostic factors that appeared in the previous studies to construct a more comprehensive prognosis model for patients with T4a GC, which also meets the requirements of precision medicine. Second, most of the studies were based on a single-centered database with a limited number of patients. Compared with this, we extracted 1,129 patients from the SEER database and performed a large cohort population-based study. Third, we further stratified patients with T4a GC into high- and low-risk groups based on the median risk score, which step closer to the clinical practice and provided a detailed stratification method for a better treatment decision of surgeons. Finally, in the construction and validation of the nomograms, ROC curves, calibration curves, and DCA analysis were used to estimate the performance of the nomograms, such a comprehensive analysis is also an important improvement in our research.

In our study, surgery, radiotherapy, and chemotherapy were associated with a better prognosis. Radical resection is still an important method for the treatment of GC, but there were also studies with opposite views. According to the research of Martin et al. (19), for patients with T4 GC, resection of two or more adjacent organs for achieving R0 resection will increase the risk of postoperative morbidity and mortality, but that does not contradict our conclusion. In the research of Li et al. (9), patients who underwent R0 resection have a higher survival rate than that of R1/2 without radical resection, and most of the postoperative morbidities were mild complications that only need short-term medical intervention, so radical treatment including resection of multiple adjacent organs is still recommended. Since patients with GC with peritoneal invasion still inevitably face the risk of metastasis and recurrence after resection, Dikken et al. (20) found that after D1 surgery, the addition of chemoradiotherapy had a major impact on reducing recurrence. The same conclusion was also introduced by Ozden et al. (21). In the recent years, the concept of adjuvant therapy has gradually entered the scene. Theoretically, if chemotherapy can reduce the size of GC tissue thus achieving the purpose of downgrading, the possibility of complete resection will be promoted, and the micrometastasis will also be eliminated to a certain extent. Yoshikawa et al. (22) also demonstrated that preoperative adjuvant chemotherapy may have a potential role in promoting the survival of patients with T3/T4 GC. In conclusion, for T4a GC, radical resection combined with multimodality therapy may be an effective means to improve the survival rate, but effective treatment strategies are still controversial.

It is still controversial that whether tumor size should be an important prognostic factor. Lee et al. (23) found that the size of the tumor had little correlation with survival or recurrence in patients with node-negative AGC, compared with those who emphasized the prognostic value of tumor size (24, 25). In addition, Lee et al. suggested that the confounding of GC types with significantly different prognosis may lead to their conclusion. Despite that, Deng et al. (26) have demonstrated that tumor size could enhance the survival discriminations in patients with T4a GC. Theoretically, a larger tumor size might relate to a higher biologic malignancy, for patients with T4a GC; peritoneal metastasis contributed greatly to the postoperative mortality (27), which might explain the reason why tumor size showed favorable utilization in the prognosis prediction of patients with T4a GC. In addition to the tumor size, another important prognostic factor is the histopathological type. In this study, the pathological types including SRCC, mucinous adenocarcinoma, and adenocarcinoma were considered, and the results of both univariate and multivariate analysis showed that the pathological type of T4a GC was significantly correlated with the prognosis. Among them, SRCC was associated with a worse prognosis, whereas the prognosis of patients with mucinous adenocarcinoma was relatively better. In the study of Yang et al. (28), SRCC accompanied by “migratory cancerous embolus of lymphatic” showed a high malignant phenotype, and the 5-year survival rate was only 15.9%, whereas that rate of mucinous gastric carcinoma (MGC) was only 19.4%. Although different pathological types play different roles in the formation of malignant phenotypes, the prognosis of these two types is not favorable. Thus, early detection and precise prognosis prediction are important in forming treatment strategies and prolong the survival of patients. Bozkaya et al. (29) also showed that patients with SRCC had worse prognosis and survival probability than MGC, and the degree of lymph node invasion was significantly correlated with OS, which was consistent with this study. Although, there were few studies discussed the difference between adenocarcinoma and the above two pathological types, it is undeniable that the malignant degree of tumor cells plays an important role in the prognosis of patients with T4a GC.

Several limitations of this study need to be acknowledged. First, since this study is a retrospective study based on the SEER database, information and selection bias might have been introduced. Second, the SEER database does not provide the access to detailed clinical information, tumor depth, metastatic sites, and operation methods were not documented, with detailed treatment strategy information, and the exploration of the prognosis of different treatment strategy might be possible. Additionally, the reason why some patients did not undergo surgery is unclear. Third, the nomogram constructed in this study could only predict OS and CSS to a maximum of 5 years due to the limited follow-up period. Despite these limitations, this is still a large population-based study that investigated the prognostic factors of patients with T4a GC, and the favorable clinical utilization of the nomogram was further confirmed.

## Conclusions

This study identified age, tumor size, race, histologic type, N stage, surgery status, radiotherapy, and chemotherapy as prognostic factors for both OS and CSS in patients with T4a GC. These factors were incorporated to construct the nomograms, and the nomograms may assist with patient assessments and help physicians to make an appropriate clinical decision.

## Data Availability Statement

The original contributions presented in the study are included in the article/Supplementary Material, further inquiries can be directed to the corresponding author.

## Author Contributions

ZH, YD, and ZZ conducted the experiments. ZH, YD, and HM analyzed the data. ZZ wrote the manuscript. ZN contributed to the conception of the present research and was responsible for approving the version before publication. All authors participated in the design, manuscript revision, read, and approved the submitted version.

## Funding

The research was supported by the National College Students Innovation and Entrepreneurship Training Program (202010343034) and Zhejiang Province Science and Technology Plan Research and Xinmiao Talent Program (2019R413001 and 2020R413018).

## Conflict of Interest

The authors declare that the research was conducted in the absence of any commercial or financial relationships that could be construed as a potential conflict of interest.

## Publisher's Note

All claims expressed in this article are solely those of the authors and do not necessarily represent those of their affiliated organizations, or those of the publisher, the editors and the reviewers. Any product that may be evaluated in this article, or claim that may be made by its manufacturer, is not guaranteed or endorsed by the publisher.
