# Supplementary material for: Incidence, Risk Factors and Prognosis of T4a Gastric Cancer: A Population-Based Study
Source: Front Med (Lausanne). 2022 Jan 5;8:767904. doi: 10.3389/fmed.2021.767904 (PMC8766749; doi:10.3389/fmed.2021.767904)
Supplement: Supplementary file 1 [file Data_Sheet_1.docx]

**Supplementary Table 1.** Uni and multivariate Cox analyses for the Overall Survival of whole T4aN0-3M0 gastric cancer patients in the whole cohort.

|  | Univariate Cox | | |  | Multivariate Cox | | |
| --- | --- | --- | --- | --- | --- | --- | --- |
|  | HR | 95% CI | P |  | HR | 95%CI | P |
| Age |  |  |  |  |  |  |  |
| Low | Reference | | | | | | |
| Middle | **1.337** | **1.139-1.568** | **0.000** |  | **1.297** | **1.101-1.527** | **0.002** |
| High | **2.068** | **1.708-2.504** | **0.000** |  | **1.579** | **1.276-1.954** | **0.000** |
| Race |  |  |  |  |  |  |  |
| White | Reference | | | | | | |
| Black | 1.055 | 0.858-1.297 | 0.612 |  | 1.129 | 0.915-1.393 | 0.258 |
| Other | **0.741** | **0.621-0.883** | **0.001** |  | **0.717** | **0.600-0.858** | **0.000** |
| Sex(Male) | 1.009 | 0.939-1.083 | 0.809 |  |  |  |  |
| Histologic type |  |  |  |  |  |  |  |
| Adenocarcinoma | Reference | | | | | | |
| Mucinous adenocarcinoma | 0.771 | 0.481-1.233 | 0.277 |  | 0.807 | 0.502-1.296 | 0.375 |
| Signet ring cell carcinoma | **1.17** | **1.005-1.363** | **0.043** |  | 1.102 | 0.938-1.295 | 0.235 |
| Size |  |  |  |  |  |  |  |
| Low | Reference | | | | | | |
| Middle | **1.282** | **1.051-1.564** | **0.014** |  | 1.202 | 0.98-1.474 | 0.077 |
| High | **2.006** | **1.571-2.561** | **0.000** |  | **1.650** | **1.28-2.125** | **0.000** |
| Grade |  |  |  |  |  |  |  |
| I | Reference | | | | | | |
| II | 1.125 | 0.523-2.421 | 0.764 |  | 0.746 | 0.344-1.616 | 0.458 |
| III | 1.618 | 0.768-3.410 | 0.206 |  | 1.104 | 0.519-2.348 | 0.797 |
| IV | **2.845** | **1.208-6.702** | **0.017** |  | 1.698 | 0.714-4.042 | 0.231 |
| N stage |  |  |  |  |  |  |  |
| N0 | Reference | | | | | | |
| N1 | **1.405** | **1.095-1.804** | **0.008** |  | **1.530** | **1.186-1.972** | **0.001** |
| N2 | 1.258 | 0.980-1.614 | 0.072 |  | **1.501** | **1.162-1.938** | **0.002** |
| N3 | **1.949** | **1.568-2.422** | **0.000** |  | **2.111** | **1.678-2.657** | **0.000** |
| Surgery performed | **0.392** | **0.295-0.522** | **0.000** |  | **0.307** | **0.228-0.412** | **0.000** |
| Radiotherapy performed | **0.608** | **0.525-0.704** | **0.000** |  | **0.772** | **0.651-0.916** | **0.003** |
| Chemotherapy performed | **0.513** | **0.444-0.594** | **0.000** |  | **0.579** | **0.482-0.695** | **0.000** |
| Insurance(Yes) | 0.775 | 0.538-1.117 | 0.172 |  |  |  |  |
| Married(Yes) | **0.721** | **0.625-0.832** | **0.000** |  | 0.870 | 0.749-1.009 | 0.066 |

**Supplementary Table 2.** Multivariate competing risk model analysis of CSS for each variable in T4aN0-3M0 gastric cancer patients in the whole cohort.

|  | HR | 95%CI | P |
| --- | --- | --- | --- |
| Age |  |  |  |
| Low | Reference | | |
| Middle | 1.080 | 0.910-1.282 | 0.380 |
| High | **1.310** | **1.030-1.667** | **0.028** |
| Race |  |  |  |
| White | Reference | | |
| Black | 0.948 | 0.753-1.192 | 0.650 |
| Other | **0.735** | **0.611-0.885** | **0.001** |
| Sex (Male) | 1.003 | 0.857-1.173 | 0.970 |
| Histologic type |  |  |  |
| Adenocarcinoma | Reference | | |
| Mucinous adenocarcinoma | 0.783 | 0.454-1.349 | 0.380 |
| Signet ring cell carcinoma | 1.120 | 0.943-1.330 | 0.200 |
| Size |  |  |  |
| Low | Reference | | |
| Middle | 1.237 | 0.998-1.532 | 0.052 |
| High | **1.779** | **1.364-2.319** | **0.000** |
| Grade |  |  |  |
| I | Reference | | |
| II | 0.789 | 0.341-1.824 | 0.580 |
| III | 1.114 | 0.489-2.536 | 0.800 |
| IV | 2.150 | 0.869-5.317 | 0.098 |
| N stage |  |  |  |
| N0 | Reference | | |
| N1 | **1.500** | **1.136-1.981** | **0.004** |
| N2 | **1.569** | **1.193-2.063** | **0.001** |
| N3 | **2.054** | **1.596-2.642** | **0.000** |
| Surgery performed | **0.338** | **0.238-0.478** | **0.000** |
| Radiotherapy performed | **0.733** | **0.619-0.868** | **0.000** |
| Chemotherapy performed | **0.671** | **0.552-0.815** | **0.000** |
| Insurance(Yes) | 0.710 | 0.474-1.064 | 0.097 |
| Married(Yes) | 0.899 | 0.763-1.060 | 0.210 |
